# Regulation of cortical stability by RhoGEF3 in mitotic Sensory Organ Precursor cells in *Drosophila*

**DOI:** 10.1242/bio.026641

**Published:** 2017-11-03

**Authors:** Lydie Couturier, Khalil Mazouni, Fred Bernard, Charlotte Besson, Elodie Reynaud, François Schweisguth

**Affiliations:** 1 Institut Pasteur, Department of Developmental and Stem Cell Biology, F-75015 Paris, France; 2 CNRS, UMR3738, F-75015 Paris, France; 3 Université Pierre et Marie Curie, Cellule Pasteur UPMC, rue du Dr Roux, 75015 Paris, France

**Keywords:** Actin, Cell division, Cortical stability, *Drosophila*, RhoGEF3

## Abstract

In epithelia, mitotic cells round up and push against their neighbors to divide. Mitotic rounding results from increased assembly of F-actin and cortical recruitment of Myosin II, leading to increased cortical stability. Whether this process is developmentally regulated is not well known. Here, we examined the regulation of cortical stability in Sensory Organ Precursor cells (SOPs) in the *Drosophila* pupal notum. SOPs differed in apical shape and actomyosin dynamics from their epidermal neighbors prior to division, and appeared to have a more rigid cortex at mitosis. We identified RhoGEF3 as an actin regulator expressed at higher levels in SOPs, and showed that RhoGEF3 had *in vitro* GTPase Exchange Factor (GEF) activity for Cdc42. Additionally, RhoGEF3 genetically interacted with both Cdc42 and Rac1 when overexpressed in the fly eye. Using a null *RhoGEF3* mutation generated by CRISPR-mediated homologous recombination, we showed using live imaging that the *RhoGEF3* gene, despite being dispensable for normal development, contributed to cortical stability in dividing SOPs. We therefore suggest that cortical stability is developmentally regulated in dividing SOPs of the fly notum.

## Abstract (precis)

**Summary:** RhoGEF3 is a developmentally regulated Cdc42 GEF that contributes to cortical stability during asymmetric divisions of Sensory Organ Precursor cells in *Drosophila*.

## INTRODUCTION

Epithelia function as protective and selective barriers between the external world and the body interior. In proliferating epithelia, cells adopt various shapes and dimensions at interphase: cells can be elongated in columnar and pseudostratified epithelia, or be flat in squamous epithelia. Despite these morphological differences, cells adopt at mitosis a spherical shape (Cadart et al., 2014; Lancaster et al., 2013; Maddox and Burridge, 2003; Ramanathan et al., 2015; Son et al., 2015; Stewart et al., 2011). This mitotic cell rounding is important for the efficient formation of a bipolar spindle and organization of a metaphase plate (Champion et al., 2017; Lancaster et al., 2013). Thus, upon mitosis, epithelial cells push on their neighbors as they round up. In columnar epithelia, mitotic cells can escape compression forces exerted by neighboring cells by dividing at the apical surface (Lee and Norden, 2013). However, in cuboidal epithelia facing a rigid apical substrate, such as the fly notum that is covered by a rigid pre-cuticle, mitotic cells round-up by pushing on their neighbors. Thus, increased cortical rigidity at mitosis may contribute to proper spindle geometry in cells growing in a mechanically constrained environment (Cadart et al., 2014; Cattin et al., 2015). At the molecular level, increased cortical rigidity at mitosis involves the cortical recruitment of MyoII and assembly of F-actin at the cell cortex downstream of the Rho-kinase (Maddox and Burridge, 2003) and mitotic kinases (Ramanathan et al., 2015; Rosa et al., 2015). In *Drosophila*, a RhoGEF known as Pebble (Pbl; Ect2 in mammals) acts as a Cdc42 GEF to regulate the formation of an isotropic actin cortex (Oceguera-Yanez et al., 2005; Rosa et al., 2015). Additionally, Moesin and its activating kinase Slik are also required for proper cortical rigidity and cell rounding (Carreno et al., 2008; Kunda et al., 2008). Thus, the mechanisms regulating cortical rigidity at mitosis and the relevance of mitotic rounding for optimal division geometry are now well established. Because most growing epithelia comprise different cell types, cell-specific regulation of mitotic rounding might exist. Whether mitotic rounding is developmentally regulated remains to be investigated.

The developing notum of *Drosophila* is an excellent model to study *in vivo* epithelial cell division and the role of mitotic spindle orientation in morphogenesis and cell fate (Bosveld et al., 2016; Gho and Schweisguth, 1998; Schweisguth, 2015). The pupal notum is a single-layered epithelium that produces the dorsal thorax of adult flies. This epithelium comprises two types of cell, the sensory organ cells and the epidermal cells. Epidermal cells divide with a random orientation (Besson et al., 2015; Bosveld et al., 2016; Gho et al., 1999). By contrast, Sensory Organ Precursor cells (SOPs) divide asymmetrically along the anterior-posterior axis of the fly body to produce an anterior pIIb cell and a posterior pIIa cell (Gho et al., 1999; Gho and Schweisguth, 1998). The orientation of the mitotic spindle is regulated by Frizzled-mediated Planar Cell Polarity (PCP) signaling (Bellaïche et al., 2001a; David et al., 2005; Gho and Schweisguth, 1998; Gomes et al., 2009; Roegiers et al., 2001), whereas the binary pIIa/pIIb decision depends on the unequal segregation of two Notch regulators, Numb and Neuralized (Le Borgne and Schweisguth, 2003; Rhyu et al., 1994; Schweisguth, 2015). Thus, SOPs respond to PCP cues and orient its spindle in response to these global cues in face of more local compression forces, e.g. resulting from the division of neighboring epidermal cells. Considering the importance of mitotic rounding for cells dividing in a crowded environment, we wondered whether the dynamics of actin and myosin might be regulated in a SOP-specific manner.

Here, we found that SOPs and epidermal cells differed in actomyosin dynamics at interphase and that SOPs appeared to be more circular at mitosis than epidermal cells. We identified RhoGEF3 as a protein expressed at higher levels in SOPs and showed that RhoGEF3 had *in vitro* GEF activity for Cdc42. Moreover, RhoGEF3 was found to genetically interact with Cdc42 and Rac1 in a gain-of-function assay. We generated a null allele of the *RhoGEF3* gene and observed that the activity of RhoGEF3 gene was largely dispensable for viability and development. However, loss of *RhoGEF3* activity increased cortical instabilities in SOPs, suggesting that RhoGEF3 contributed to cortical stability in mitotic SOPs.

## RESULTS

### Apical shape and actomyosin dynamics in SOPs

**Fig. 1.**
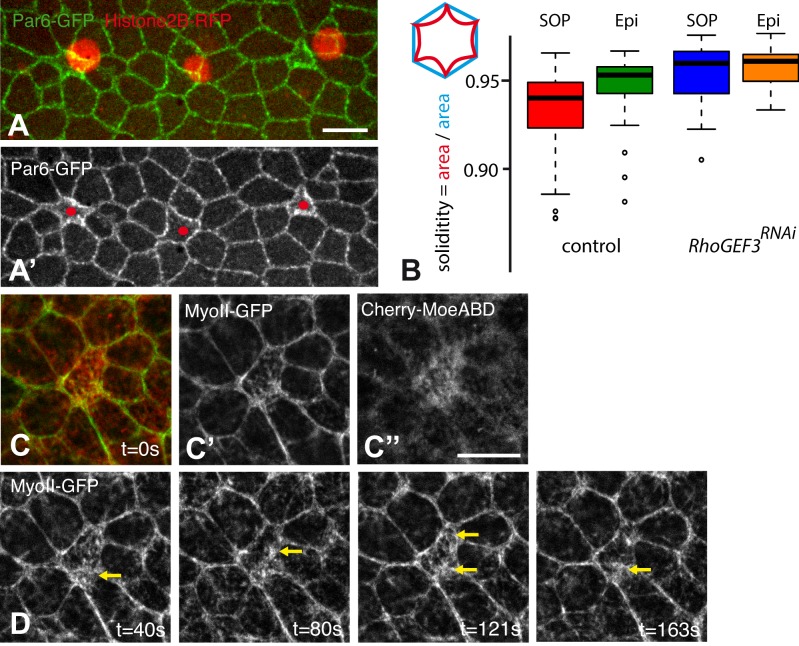
**Cell-specific differences in shape and actomyosin at interphase.** (A,A′) Snapshot from a Par6-GFP (green) movie (*n*>20 movies) showing that SOPs (Histone2B-RFP, red) can be recognized based on their concave edges in the pupal notum at 15 h after puparium formation (APF). (B) Boxplot analysis of solidity in wild-type SOPs (red, *n*=49) and epidermal cells (Epi; green, *n*=46) as well as *RhoGEF3^RNAi^* SOPs (blue, *n*=30) and epidermal cells (Epi; orange, *n*=26) using GFP-Baz as a cortical marker. A significant difference in solidity was observed between wild-type SOPs and epidermal cells (*P*=0.0007; Wilcoxon test). Silencing of the *RhoGEF3* gene abolished this difference (*P*=0.7). Solidity is defined by the ratio between the actual area of the shape region (segmented contour, red in the diagram at the top left) and the convex hull of the shape (blue). (C-D) Myosin (MyoII-GFP, green, C,C′,D) and F-actin (Cherry-MoeABD, red in C,C″) were detected at the medial-apical cortex of SOPs in the notum of living pupae at 16 h APF (*n*=8 movies). Snapshots showing pulses and/or waves of MyoII-GFP recruitment at the medial-apical cortex (see Movie 1). Time (t) is in seconds (s; see panel C′ for t=0). Scale bars: 5 µm.

While studying the polar distribution of Par6 and Baz in the single-layered epithelium that will form the dorsal thorax (Besson et al., 2015), we noticed that SOPs could be recognized from other non-SOP epithelial cells by their reduced apical area and their concave edges, leading to a characteristic inward-curving shape (Fig. 1A,A′). To measure the extent to which the apical area is concave, we used ‘solidity’ as a shape descriptor (‘solidity’ measures the ratio between the area of the apical cortex region and the convex hull of the shape; see diagram in Fig. 1B). Using the cell contours extracted from the time-lapse movies reported in Besson et al. (2015), we characterized the shape of the apical surface in SOPs and epidermal cells at interphase and found a significant difference in ‘solidity’ between these two types of cells prior to mitosis (Fig. 1B). Since epithelial cells are in a dynamic mechanical equilibrium at the level of their apical junction (Heisenberg and Bellaïche, 2013; Lecuit et al., 2011), this difference in cell shape suggested differences in mechanical properties between SOPs and their neighbors.

Within each cell, a contractile actomyosin meshwork generates active forces. High contractile activity along a given cell edge results in edge straightening and shortening. Conversely, increased contractility of medial actomyosin produces a centripetal flow associated with inward pulling forces exerted on cell edges (Heisenberg and Bellaïche, 2013; Martin et al., 2010, 2009; Martin and Goldstein, 2014). Accordingly, the observed inward-curving of the SOP edges indicated cell-specific differences in actomyosin dynamics between SOPs and their neighboring epidermal cells. To test this possibility, we examined the distribution of F-actin, using the Actin Binding Domain (ABD) of Moesin (Moe) fused to mCherry (Cherry-MoeABD), and Myosin II (MyoII), using a MyoII-GFP reporter. Live imaging of F-actin revealed an accumulation of F-actin at the apical surface of SOPs (Fig. 1C-C″). This observation confirmed earlier findings showing that F-actin localized differentially in SOPs and epidermal cells to promote the formation of microvilli in SOPs and in their progeny cells (Rajan et al., 2009). Here, we found that MyoII was recruited to the apical medial cortex, together with F-actin, in SOPs (Fig. 1C-C″). Moreover, contractile pulses and foci of MyoII were observed at the medial cortex of SOPs (Fig. 1D; Movie 1). By contrast, MyoII was mostly junctional in neighboring non-SOP cells and appeared to form supracellular junctional cables. Thus, our data revealed a change in actomyosin distribution in SOPs characterized by an increase in the medial-apical pool of contractile actomyosin. Of note, no changes in E-Cadherin (E-Cad), Armadillo (Arm, fly β-catenin) and p120catenin levels were observed (not shown). We conclude that adoption of the SOP fate led to cell-specific differences in actomyosin organization prior to asymmetric cell division.

### Actomyosin distribution in dividing SOPs

**Fig. 2.**
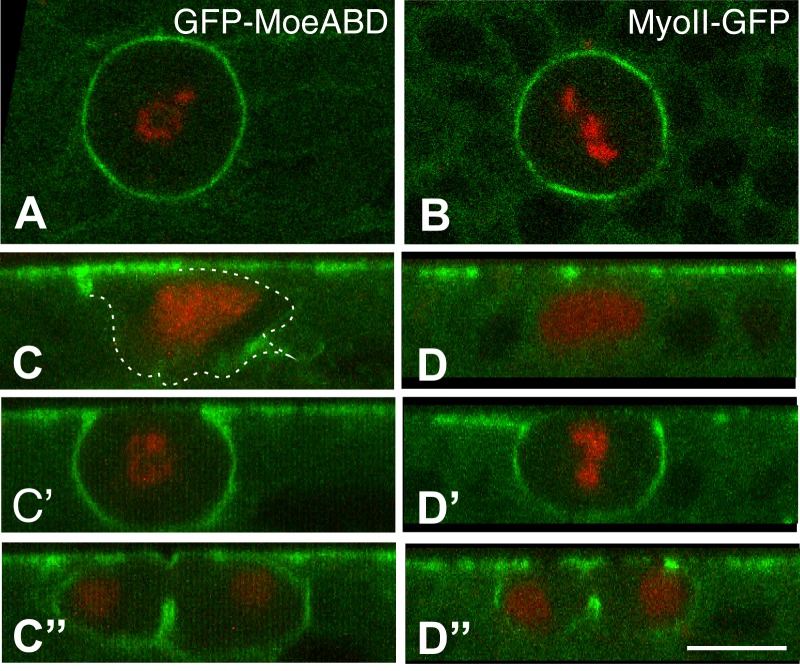
**Cell rounding and cortical recruitment of F-actin and MyoII in mitotic SOPs.** (A-D′) Snapshots from GFP-MoeABD (F-actin, green; A, x,y view and C-C″, x,z views) and MoyII-GFP (Myosin, green; B x,y view and D-D″, x,z views) movies (*n*=6 for each genotype). SOPs were marked by the Histone2B-RFP (red) expressed under the *neur* promoter. SOPs exhibited irregular shapes prior to mitosis (Georgiou and Baum, 2010) (C,D) and rounded up at mitosis (A,C′,B,D′) prior to cytokinesis (C″,D″; note the accumulation of F-actin and MyoII along the ingressing membrane and at the cytokinetic ring, respectively). Scale bar: 5 µm.

We next studied the distribution of actomyosin in mitotic SOPs. Upon entering into mitosis, SOPs became spherical and this cell rounding correlated with the redistribution of actomyosin all around the cell to presumably produce a rigid cortex at prometaphase (Founounou et al., 2013; Lancaster et al., 2013) (Fig. 2A-D″). At anaphase, asymmetric cytokinesis was observed, with the plasma membrane ingressing more rapidly on the basal side as described earlier (Founounou et al., 2013; Guillot and Lecuit, 2013; Herszterg et al., 2013; Morais-de-Sá and Sunkel, 2013). Additionally, mitotic SOPs appeared to be less deformable than mitotic epidermal cells: live imaging indicated that SOPs were more circular at mitosis than epidermal cells (circularity values measured within the plane of the epithelium: 0.941±0.004 in SOPs versus 0.902±0.021 in epidermal cells; *t*-test, *P*=4×10^−9^). This suggested that mitotic SOPs might more efficiently push on their neighbors than mitotic epidermal cells. This might in turn suggest that cortical rigidity at mitosis could be higher in SOPs than in epidermal cells.

### RhoGEF3 is expressed at higher levels in SOPs

We next investigated how cortical rigidity might be regulated in SOPs. A previous study has shown that the accumulation of F-actin and MyoII at the cell cortex in mitosis required the activity of a formin, Diaphanous (Dia), acting downstream of Cdc42 (Rosa et al., 2015). This finding raised the possibility that increased cortical rigidity in SOPs might result from increased activity of Cdc42 and/or of its downstream effectors. Interestingly, RNAseq analysis of pools of SOPs and epidermal that had been individually microdissected from fixed nota showed that a 1.8-fold increase in *RhoGEF3* transcript levels in SOPs (*P*=0.004; K.M., unpublished). Despite its name, the *RhoGEF3* gene may encode a GTPase Exchange Factor (GEF) for Cdc42/Rac, rather than for Rho (Greenberg and Hatini, 2011). Indeed, its closest mammalian ortholog, known as ARHGEF4 or ASEF, was shown to display GEF activity towards Cdc42 and Rac, but not Rho (Kawasaki et al., 2007, 2003, 2000). Moreover, analysis of the formation of multicellular capsules around parasitoid wasp eggs in fly larvae identified a requirement for Dia, Cdc42/Rac and RhoGEF3, whereas the activities of Rho1 and of its GEFs Pebble and RhoGEF2 were dispensable (Howell et al., 2012). These data are therefore consistent with RhoGEF3 acting as a Cdc42/Rac GEF, possibly upstream of Dia, for the regulation of actin in immune cells. We therefore decided to investigate further the role of RhoGEF3 in SOPs.

**Fig. 3.**
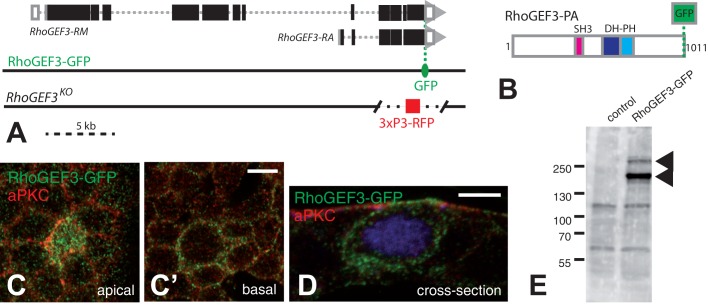
**SOP-specific expression of RhoGEF3.** (A) Genomic organization of the *RhoGEF3* locus showing only two transcript isoforms (RA and RM). Exons are shown as boxes (ORF in black). The position of GFP (green) in the RhoGEF3-GFP BAC is indicated. All isoforms are tagged. The position of the deletion in the *RhoGEF3^KO^* flies produced by CRISPR-HR is indicated: most of the sequence encoding the RhoGEF3-PA was deleted. The 3xP3-RFP selection marker was inserted at the position of the deleted segment. (B) Domain structure of RhoGEF3-PA, encoded by the RA transcript isoform in A. The catalytic DH-PH domains (blue) are shown along with an SH3 domain (magenta). GFP (green) was fused at the C-terminus. (C-D) RhoGEF3-GFP (green; aPKC, red) was detected at higher levels at the cortex of SOPs (Senseless, blue in D, showing a transverse view taken from a sectioned notum; *n*=12). (E) Western blot analysis of RhoGEF3-GFP in larval extracts prepared from brain-disc complexes showed both long (∼350 kDa) and short (∼110 kDa) protein isoforms are present in these imaginal tissues. These isoforms might be produced from long (RM-like) and short (RA-like) transcript isoforms, respectively. The blot shown here is representative of the results from two experiments. Scale bars: 5 µm.

To further study the expression of the RhoGEF3 protein in the notum, we used BAC recombineering to generate a RhoGEF3-GFP transgene. The *RhoGEF3* gene encodes multiple isoforms and because it is not known which of these isoforms are expressed in the pupal notum, we inserted GFP at the non-conserved C-terminus of RhoGEF3 that is shared by all predicted isoforms to produce RhoGEF3-GFP (Fig. 3A,B). Western blot analysis of larval brain-disc complexes indicated that both long and short isoforms of RhoGEF3 were present in these tissues (Fig. 3E). Furthermore, consistent with our RNAseq data, we found that RhoGEF3-GFP was present in all cells of the pupal notum at a low level and that SOPs exhibited higher levels of RhoGEF3-GFP accumulation (Fig. 3C,C′). At mitosis, RhoGEF3-GFP distributed all around the cortex (Fig. 3D). Thus, RhoGEF3 is expressed at higher levels in SOPs.

Our RNAseq analysis of SOPs identified several other actin regulators, including the *Daam* gene, one of the six formin family genes of *Drosophila* (3.2-fold increase in mRNA levels in SOPs, *P*=2×10^−9^; K.M., unpublished). Using a *Daam^GFP^* knock-in allele produced by CRISPR-mediated Homologous Recombination (HR), we confirmed that Daam was expressed at higher levels in SOPs and, like Dia, localized all around the cortex in mitotic SOPs (Fig. S1A-D′). While these expression and localization data suggested that Daam might regulate cortical stability in mitotic SOPs, the silencing of the *Daam* gene had no detectable effect on cell shape and cortical stability (data not shown). We therefore focused our analysis on the role of RhoGEF3 in cortical stability.

### RhoGEF3 has *in vitro* Cdc42 GEF activity

**Fig. 4.**
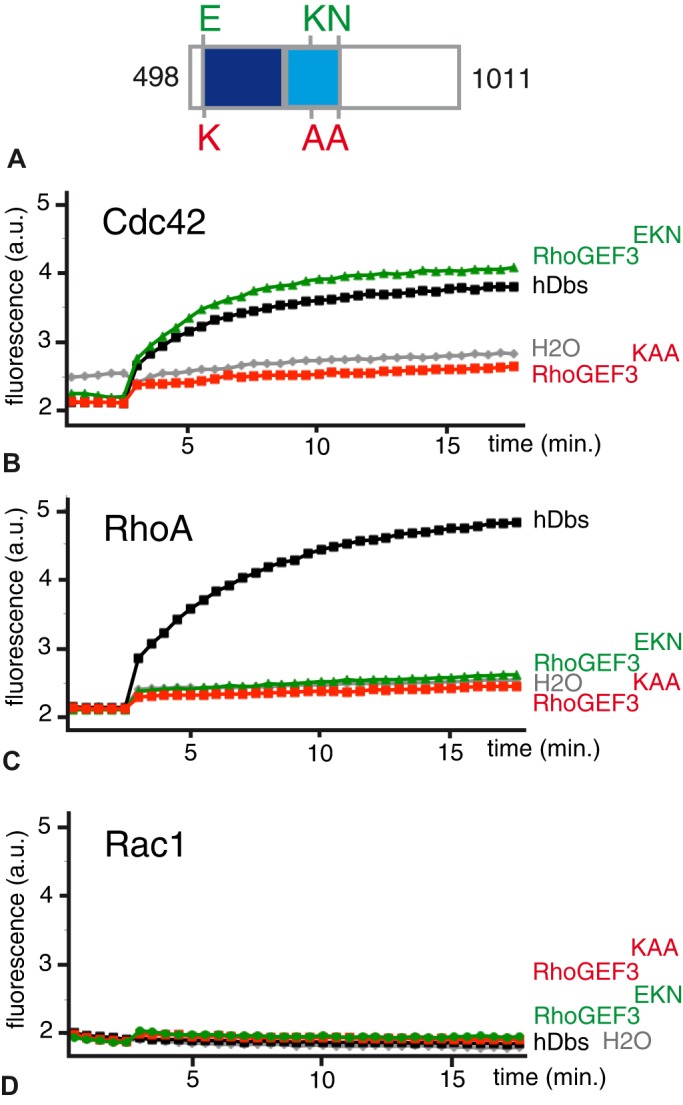
**Analysis of the *in vitro* GEF activity of RhoGEF3.** (A) Domain structure of the RhoGEF3^EKN^ (wild-type) and RhoGEF^KAA^ (catalytically-dead) fragments produced in *E. coli* and tested for *in vitro* GEF activity. (B-D) The RhoGEF3 exchange activity for Cdc42 (B), RhoA (C) and Rac1 (D) was measured *in vitro* as an increase over time of the fluorescence resulting from the incorporation of mant-GTP, a fluorescent nucleotide analog, into the binding pocket of these GTPases. A fragment of human Dbs served as a positive control for Cdc42 and Rho GEF activity, whereas RhoGEF^KAA^ served as a negative control for RhoGEF3^EKN^. The graphs shown here are representative of the results obtained in this experiment, repeated three times.

We first studied the GEF activity of RhoGEF3 using an *in vitro* GEF assay. The activity of a 60 kDa fragment of RhoGEF3 that is present in all isoforms and which encodes the predicted GEF domain (RhoGEF3^EKN^) (Fig. 4A) was produced in *E. coli* as a GST-fusion protein, purified and tested against Cdc42, Rac1 and RhoA. As a negative control, we used a version of RhoGEF3 that harbored mutations at conserved residues that are known to be important for the GEF catalytic activity of Lcp/p115 RhoGEF (Dubash et al., 2007) and GEF-H1 (Cullis et al., 2014). Specifically, the conserved E526 (numbering as in RhoGEF3-PA) was mutated into a Lysine (K) as in GEF-H1 (Cullis et al., 2014) and the conserved K677 and N726 residues were mutated into Alanine (A) as in Lcp/p115 RhoGEF (Dubash et al., 2007). The triple mutant protein used as a negative control in the GEF assay was referred to here as RhoGEF3^KAA^ (Fig. 4A). We used a fluorescent nucleotide analog (mant-GTP) to follow nucleotide exchange on purified Cdc42, Rac1 and RhoA. Since the binding of mant-GTP to the nucleotide binding pocket of a GTPase results in fluorescence increase (Leonard et al., 1994), an increase in fluorescence intensity indicated nucleotide exchange by the GTPase. Using this *in vitro* assay, we found that RhoGEF3^EKN^, but not RhoGEF3^KAA^, displayed GEF activity towards Cdc42, but not towards Rac or Rho (Fig. 4B-D). This result indicated that RhoGEF3 may act as a GEF for Cdc42.

### RhoGEF3 genetically interacts with Cdc42 and Rac1

**Fig. 5.**
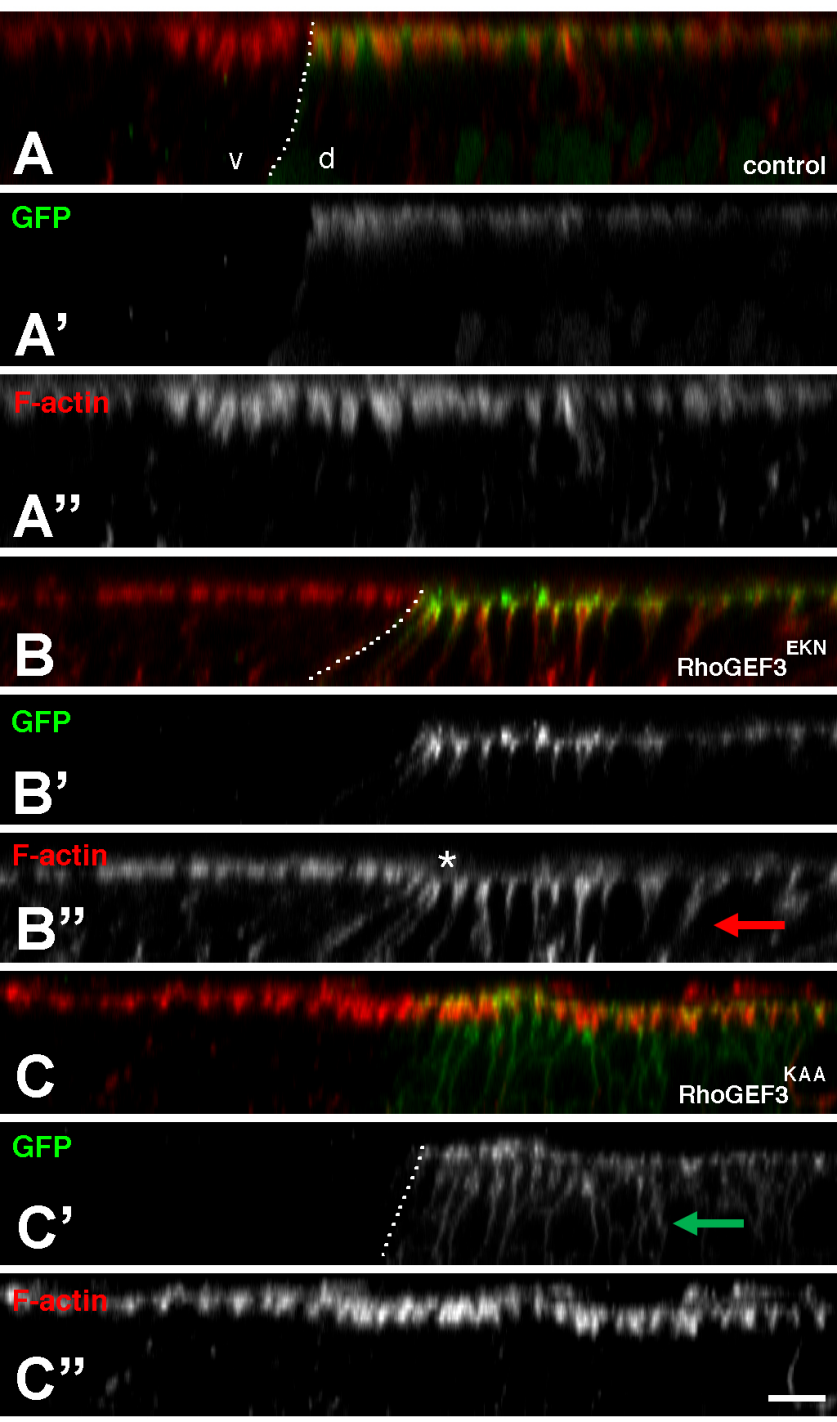
**Overexpressed RhoGEF3 regulates F-actin distribution in a GEF-dependent manner.** Cross-section views of third instar wing imaging discs showing the distribution of F-actin (phalloidin, red; apical is up). In control discs (A-A″; *ap-Gal4 Gal80ts UAS-nlsGFP* at 25°C), F-actin localizes apically in both ventral (v) and dorsal (d) cells (the limit between d and v cells is indicated with a dotted line). Overexpression of RhoGEF3^EKN^-GFP in dorsal cells (B-B″; *ap-Gal4 Gal80^ts^ UAS-RhoGEF3^EKN^GFP*) led to the ectopic accumulation of F-actin along lateral membranes (red arrow in B″). Note also the shortening of the v cells, resulting in a depressed area (asterisk) and occasional folds (not shown). These effects were dependent on the GEF activity of RhoGEF3 as it was not seen in cells expressing the catalytically-dead RhoGEF3^KAA^ mutant (C-C″; *ap-Gal4 Gal80ts UAS-RhoGEF3^KAA^GFP*). Also, while wild-type RhoGEF3^EKN^-GFP (B,B′; green) localized apically, catalytically-dead RhoGEF3^KAA^GFP (C,C′; green) accumulated at both apical and lateral cortex (green arrow in C′). At least eight imaginal discs per genotype were scanned. Scale bar: 5 µm.

To further investigate *in vivo* the GEF activity of RhoGEF3, we generated transgenic flies expressing under the control of the UAS/Gal4 system two GFP-tagged versions of the long RhoGEF3 isoform (RhoGEF3-PL): a wild-type version (RhoGEF3^EKN^-GFP) and a version mutated at the E, K and N residues that are required for its GEF activity (RhoGEF3^KAA^-GFP). RhoGEF3^EKN^-GFP localized at the apical cortex when expressed in the dorsal cells of wing imaginal discs under the control of the ap-Gal4 driver line. Additionally, expression of active RhoGEF3 led to increased F-actin levels along the lateral membranes (Fig. 5A-B″). This lateral accumulation of F-actin appeared to correlate with a shortening of cell height and formation of epithelial folds in the dorsal part of the wing pouch (data not shown). By contrast, the catalytically dead version of RhoGEF3, RhoGEF3^KAA^-GFP, localized along both apical and lateral membranes and had no effect on F-actin distribution and epithelium shape (Fig. 5C-C″). These observations are consistent with the notion that RhoGEF3 regulates the organization of the actin cytoskeleton in a GEF-dependent manner.

**Fig. 6.**
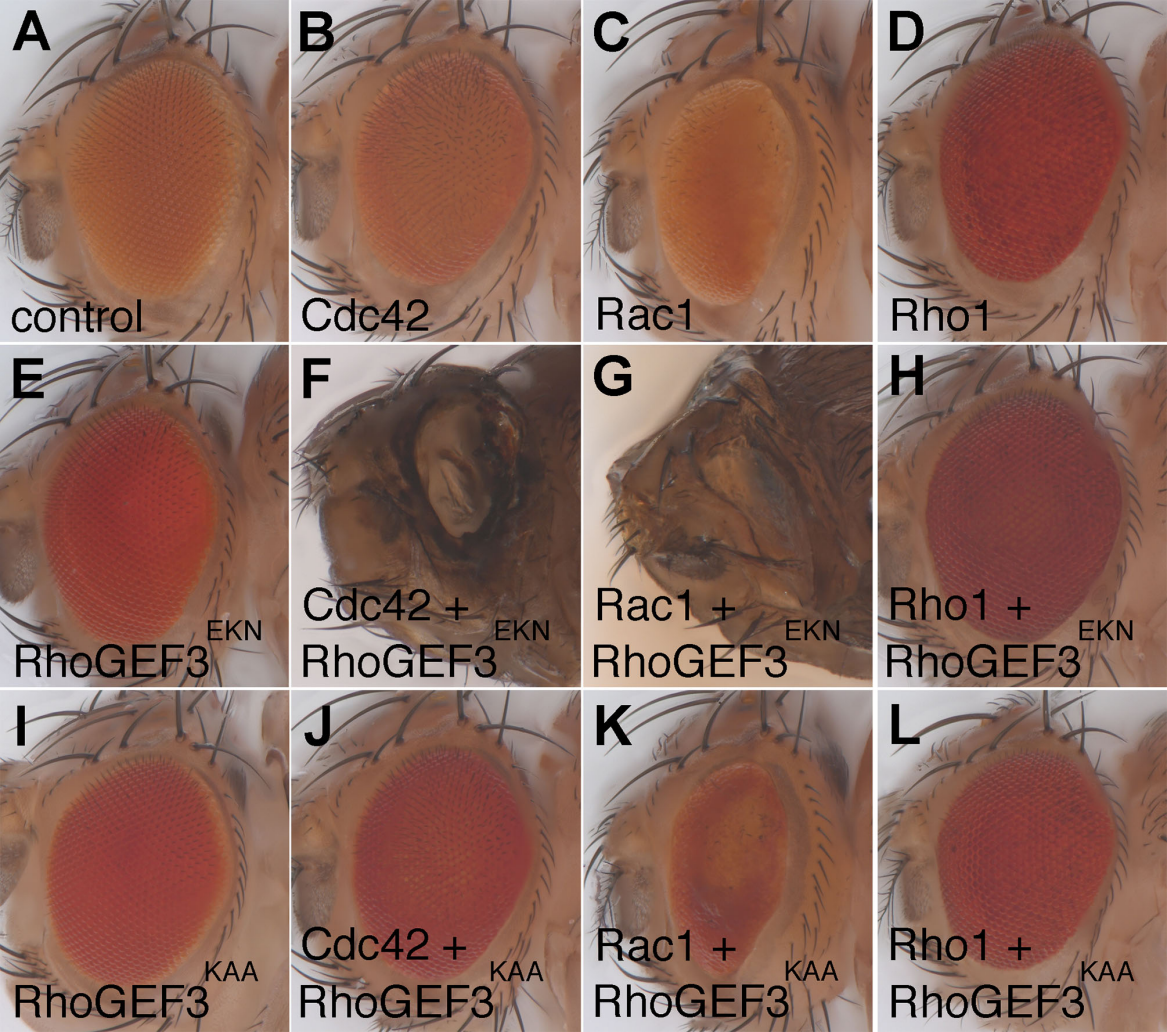
**RhoGEF3 genetically interacts with Cdc42 and Rac1.** Stereomacroscope pictures of adult compound eyes of GMR-Gal4/+ (A), GMR> Cdc42 (B), GMR> Rac1 (C), GMR> Rho1 (D), GMR> RhoGEF3^EKN^-GFP (E), GMR>RhoGEF3^EKN^-GFP+Cdc42 (F), GMR->RhoGEF3^EKN^-GFP+Rac1 (G), GMR>RhoGEF3^EKN^-GFP+Rho1 (H), GMR>RhoGEF3^KAA^-GFP (I), GMR>RhoGEF3^KAA^-GFP+Cdc42 (J), GMR>RhoGEF3^KAA^-GFP+Rac1 (K) and GMR> RhoGEF3^KAA^-GFP+Rho1 (L) flies. Due to lethality, pharate adults are shown in panels F and G. Since the activity of Gal4 is temperature-dependent, crosses were performed at 18°C (B,C,F,G,J,K) and 25°C (all other panels). Wild-type RhoGEF3 genetically interacts with Cdc42 and Rac1 (F,G) but not Rho1 (H). Interaction required an active GEF domain (J-L). At least 10 flies per genotype were scanned. Anterior is right, dorsal up.

We next addressed whether RhoGEF3 could act *in vivo* as a GEF for Cdc42, as suggested by the results of the *in vitro* GEF assay (Fig. 4). To do so, we performed genetic gain-of-function interactions in the compound fly eye. The wild-type (active) or mutant (inactive) version of GFP-tagged RhoGEF3 were expressed together with the wild-type versions of Cdc42, Rac1 or Rho1 using the UAS/Gal4 system. Expression of these three GTPases in the photoreceptor cells using the GMR-Gal4 driver produced relatively minor defects in the structure of the adult eye (Fig. 6A-D), with the strongest defects being seen with Rac1 (Hariharan et al., 1995; Nolan et al., 1998). Of note, Cdc42 and Rac1 were overexpressed at 18°C to minimize the temperature-dependent activity of Gal4, whereas Rho1 was expressed at 25°C. Likewise, expression of wild-type and mutant RhoGEF3 did not significantly alter the adult eye structure (Fig. 6E,I). However, expressing wild-type RhoGEF3 together with Cdc42 or Rac1 led to a fully penetrant late pupal lethality that was associated with a very strong eye defect in pharate adults (Fig. 6F,G). These phenotypes appeared to be similar to those reported earlier for the eye-specific expression of activated Cdc42, Rac1 and Rho1 (Benlali et al., 2000; Rangarajan et al., 1999; Schenck et al., 2003) (in our hands, driving the expression of Cdc42V12, Rac1V12 and Rho1V14 using GMR-Gal4 at 18°C led to early pupal lethality). The genetic interactions observed between RhoGEF3 and Cdc42/Rac1 were dependent on the GEF activity of RhoGEF3 as flies co-expressing Cdc42 or Rac1 with RhoGEF3^KAA^-GFP showed either no defect (Cdc42; Fig. 6J) or much reduced interaction (Rac1; Fig. 6K). Finally, no genetic interaction was observed between RhoGEF3 and Rho1 (Fig. 6H,L). Together, these data support the view that RhoGEF3 might act *in vivo* as a GEF for Cdc42 and Rac1. Thus, our *in vitro* and *in vivo* results suggest that RhoGEF3 acts as a Cdc42 GEF. Whether RhoGEF3 has also GEF activity towards Rac1 *in vivo* remains to be clarified (see discussion).

### RhoGEF3 contributes to cortical stability

**Fig. 7.**
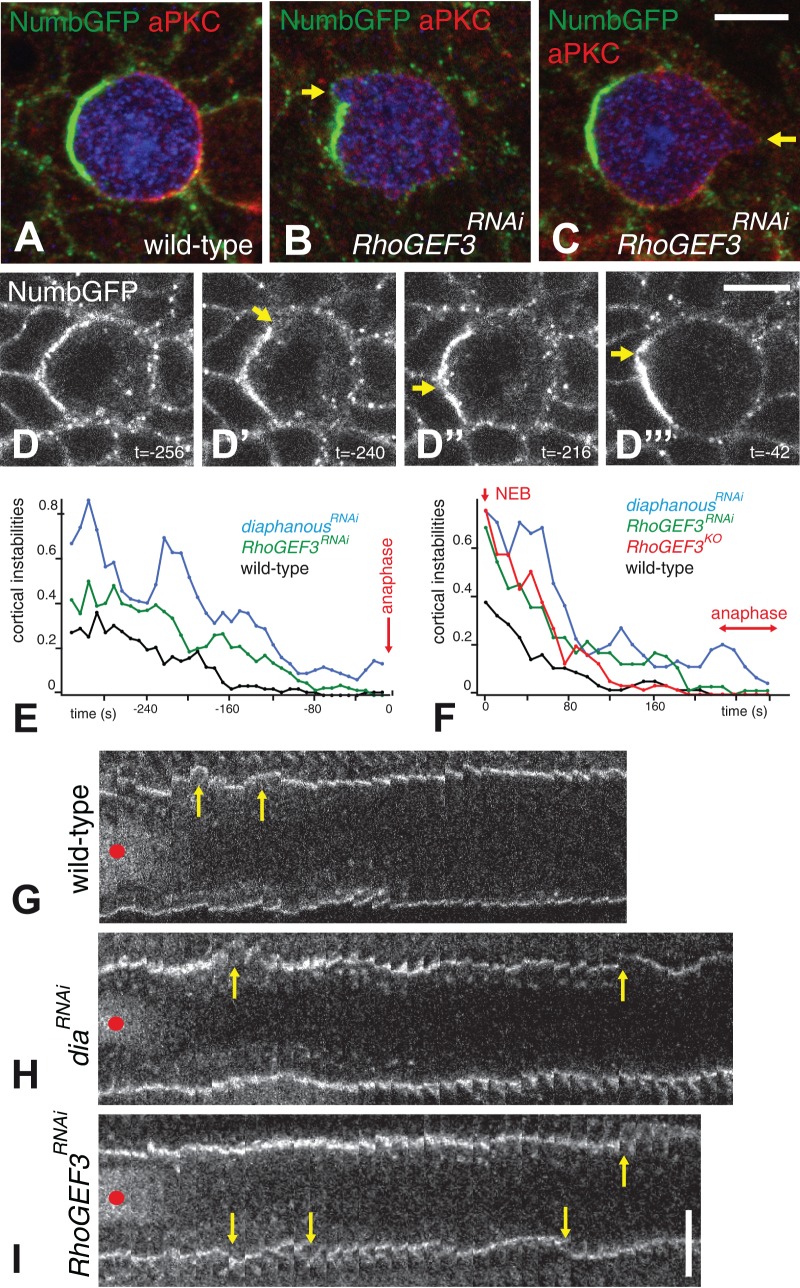
**RhoGEF3 contributes to cortex stability in mitotic SOPs.** (A-C) Cortical distribution of Numb-GFP (green) and aPKC (red) in dividing SOP (Sens, blue) in wild-type (A) and *pnr*>*RhoGEF3^RNAi^* (B,C) pupae at 16.5 h APF. Cortical instabilities were observed upon silencing of *RhoGEF3* (arrows). At least 15 SOPs per genotype were studied. (D-D‴) Live imaging of Numb-GFP in a *RhoGEF3^RNAi^* SOP. Selected snapshots showing cortical instabilities at the anterior cortex that transiently disrupted the Numb crescent (see Movie 2). Time is in s and t=0 corresponds to the metaphase-anaphase transition. (E) Quantification of the number of cortical instabilities (seen using Numb-GFP) in dividing SOPs. Cortical instabilities were scored blind in wild-type control (*n*=19), *RhoGEF3^RNAi^* (*n*=23) and *dia^RNAi^* (*n*=18) SOPs. The number of cortical instabilities at prometaphase (per cell and time interval of 8 s; time in s) increased upon the silencing of the *dia* and *RhoGEF3* gene. The metaphase-anaphase transition was chosen as t=0 (red arrow). (F) Quantification of the number of cortical instabilities seen using Spider-GFP (per cell and time interval of 8 s; time in s) in dividing SOPs. Because division time varied with genotype and most cortical instabilities were observed soon after NEB (which could be observed using Spider-GFP; see panels G-I), the NEB was chosen here as t=0 (red arrow). Cortical instabilities were scored blind in wild-type control (*n*=19), *RhoGEF3^RNAi^* (*n*=22), *RhoGEF3^KO^* (*n*=19) and *dia^RNAi^* (*n*=15) SOPs. The number of cortical instabilities at prometaphase increased upon loss of *dia* and *RhoGEF3* activities. (G-I) Kymographs of dividing Spider-GFP SOPs. The silencing of *dia* (H) and *RhoGEF3* (I) led to cortical instabilities during prometaphase (yellow arrows; the red dots indicate the nuclear Spider-GFP signal used to detect the onset of NEB; time interval is 8 s). In wild-type pupae, the cortex remained stable from NEB to anaphase in SOPs (G). Scale bars: 5 µm.

We next investigated the function of RhoGEF3 in the developing notum. To do so, we generated a null allele by creating a large deletion at the *RhoGEF3* locus using CRISPR-mediated HR (Fig. 3A). The resulting *RhoGEF3^KO^* mutant flies were semi-viable and fertile with no obvious developmental defects but appeared weak and survived poorly. In particular, no fate defects were observed in the bristle lineage (not shown). This indicated that the activity of the *RhoGEF3* gene is largely dispensable for proper fly development. To look at the possible function of the *RhoGEF3* gene in SOP asymmetric division, we performed live imaging on GFP-Baz pupae expressing a dsRNA targeting RhoGEF3 under the control of the *pnr-Gal4* driver. Although the silencing of the *RhoGEF3* gene had no effect on the posterior accumulation of GFP-Baz, or on the anterior-posterior orientation of the division (not shown), it resulted in increased solidity in SOP (Fig. 1B). This observation was consistent with a role for RhoGEF3 in actin dynamics in SOPs. Additionally, bleb-like cortical instabilities were observed in mitotic *RhoGEF3^RNAi^* SOPs in fixed nota using aPKC and Numb as markers for the posterior and anterior cortical domains, respectively (Fig. 7A-C; note that SOP asymmetry remained unaffected). Such cortical instabilities were only rarely detected in wild-type SOPs at prometaphase (Fig. 7A). This therefore raised the possibility that RhoGEF3 contributes to cortical stability in SOPs.

To better study the role of RhoGEF3 in cortical stability, we performed live imaging using Numb-GFP as a cortical marker. Because loss of *dia* has previously been reported to promote blebbing in dividing epidermal cells of the fly notum, we used *dia* as a positive control. In wild-type pupae, blebbing and/or cortical instabilities were observed basally in SOPs rounded up during prophase but were more rarely seen after nuclear envelope breakdown (NEB). By contrast, a significant number of cortical instabilities were scored at prometaphase in both *dia^RNAi^* and *RhoGEF3^RNAi^* pupae (Fig. 7D-E; scoring was performed blind). These instabilities were transient and NumbGFP segregated correctly into the anterior pIIb cell as in wild-type cells (Fig. 7D-D‴;Movie 2).

Since Numb-GFP only marked the anterior cortex, we next used a gilgamesh-GFP fusion protein, SpiderGFP, which marked the entire cortex. Low levels of SpiderGFP were detected in the nuclei during interphase and at prophase. This allowed us to precisely time the NEB. SpiderGFP movies were analyzed by scoring cortical instabilities in wild-type, *dia^RNAi^*, *RhoGEF3^RNAi^* and *RhoGEF3^KO^* mutant pupae (again, scoring was performed blind). The results showed that reduced *dia* and *RhoGEF*3 activities led to more frequent cortical instabilities after NEB in mitotic SOPs relative to control SOPs (Fig. 7F-I). We conclude that RhoGEF3 contributes to increased cortex stability in mitotic SOPs. Together, our results suggested that cortical stability at mitosis may be modulated in a cell-specific manner in the fly notum via the regulated expression of RhoGEF3.

## DISCUSSION

An Ect2/Pbl-Cdc42-Dia pathway was shown earlier to regulate mitotic rounding in epidermal cells of the fly notum (Rosa et al., 2015). This pathway was proposed to regulate the assembly of an isotropic actomyosin via the lateral spreading of Par6, hence triggering a switch upon mitotic entry from an Arp2/3-mediated actin nucleation process active at interphase to a Dia-mediated process (Rosa et al., 2015). Par6 spreads laterally at the posterior cortex in SOPs (Bellaïche et al., 2001b), but it is unclear whether a similar switch takes place in SOPs and whether additional regulatory processes might contribute to an isotropic actomyosin cortex. Here, we identified RhoGEF3 as a protein expressed at higher levels in SOPs. Using an *in vitro* assay, we found that a large 60 kDa fragment of RhoGEF3 encompassing the catalytic DH-PH domains could act as a Cdc42 GEF. Consistent with this, the co-expression of active RhoGEF3 and Cdc42 in photoreceptor cells, but not the expression of either one alone, led to pupal lethality and defective eye formation. Since similar phenotypes were observed upon expression of activated Cdc42, the strong synergy between RhoGEF3 and Cdc42 indicated that RhoGEF3 might act as *in vivo* GEF for Cdc42. Additionally, both *in vitro* Cdc42 GEF activity and *in vivo* genetic interaction were abolished upon mutation of key catalytic residues in the DH-PH domain of RhoGEF3. Thus, our data strongly suggest that RhoGEF3 can act as a Cdc42 GEF, like its mammalian homolog (Kawasaki et al., 2007, 2003, 2000). While the large 60 kDa fragment of RhoGEF3 encompassing the catalytic DH-PH domains showed no detectable GEF activity towards human Rho and Rac *in vitro*, RhoGEF3 was found to also synergize *in vivo* with Rac1 (but not Rho1). Thus, whether RhoGEF3 can also act as a Rac GEF, as shown for its mammalian homolog (Kawasaki et al., 2007, 2003, 2000), will require further investigation. In support of this view, a very recent study used a GST pulldown assay to test for direct molecular interaction between *in vitro* translated RhoGEF3 with GDP/GTP-loaded GTPases. This analysis indicated that RhoGEF3 can bind GTP-loaded Rac1 and might also regulate Cdc42 (Nakamura et al., 2017). These findings are consistent with our genetic interaction data showing a strong synergy between RhoGEF3 and Cdc42/Rac1. While it is conceivable that RhoGEF3 might regulate the localization of GTP-bound Rac1 (Nakamura et al., 2017) or act downstream of Rac1 activation as an effector of GTP-bound Rac, it is also possible that our *in vitro* assay failed to detect the Rac1 GEF activity of RhoGEF3. In summary, *Drosophila* RhoGEF3 acts as a GEF for Cdc42 and possibly Rac1, but is not a Rho GEF.

RhoGEF3 was recently reported to regulate the distribution of F-actin in a wound healing assay in embryos (Nakamura et al., 2017). Here, RhoGEF3 was found in a gain-of-function assay to alter in a GEF-dependent manner the distribution of F-actin in wing epithelial cells. This therefore suggested that RhoGEF3 has the ability to regulate F-actin distribution, presumably via Cdc42 and/or Rac1. To further examine the *in vivo* function of RhoGEF3, we used CRISPR-mediated homologous recombination to create a null deletion allele of the *RhoGEF3* gene. Our genetic analysis, however, revealed that the activity of RhoGEF3 is largely dispensable for asymmetric division of SOPs and more generally for proper development in *Drosophila*. Nevertheless, our live imaging analysis of cortical stability indicated that RhoGEF3 plays a non-essential role in the regulation of cortical stability in SOPs. Whether these cortical instabilities correlated with changes in Cdc42-dependent F-actin dynamics was not further examined. Based on these data, we speculate that RhoGEF3 contributes, in parallel to other essential Cdc42/Rac GEFs such as Ect2/Pbl (Rosa et al., 2015), to the activation of Cdc42 (and possibly Rac, but not Rho) in mitotic SOPs, thereby promoting the formation of a rigid cortex at mitosis. While this activity is non-essential for the maintenance of SOP asymmetry at mitosis, we speculate that this activity might contribute to stabilize the cortical domains of asymmetrically dividing SOPs in face of local deformations, thereby ensuring that SOPs divide asymmetrically along a stereotyped division axis independently of the behavior of their neighbors within a crowded environment (Cadart et al., 2014). This view is consistent with earlier studies showing that increased actin polymerization, downstream of the SRF transcription factor, is essential for spindle orientation and asymmetric division in the skin of the mouse embryo (Luxenburg et al., 2011). While the fly homolog of SRF was not detected as being upregulated in SOPs, several actin regulators, including RhoGEF3 and Daam, were expressed at significantly different levels in SOPs versus epidermal cells. Thus, we speculate that RhoGEF3 might be only one of several activities contributing to the SOP-specific changes in actomyosin dynamics. Future studies looking for such redundant activities might in turn further our understanding of the *in vivo* function of RhoGEF3 in the cell-specific regulation of cortical stability.

## MATERIALS AND METHODS

### Transgenes, genome engineering and flies

The RhoGEF3-GFP BAC transgene was generated using recombineering mediated gap-repair from the BAC CH322-144N20 (Venken et al., 2009, 2006). The sfGFP flanked by GVG linkers was fused in frame at the C-terminus of RhoGEF3. The resulting BAC was integrated at the M{3xP3-RFP.attP}ZH-51D site. The pUAS-RhoGEF3^EKN^-GFP and pUAS-RhoGEF3^KAA^-GFP plasmids were generated in two steps. First, the EKN-to-KAA mutations were introduced in the RhoGEF3-GFP BAC using BAC recombineering. Second, genomic fragments encoding the short isoforms, e.g. RhoGEF3-PB, were PCR-amplified from wild-type and mutated BACs and subcloned as a EcoRI-XbaI fragment into the pUAS-attB vector. The resulting UAS-RhoGEF3^EKN^-GFP and UAS-RhoGEF3 ^KAA^-GFP plasmids were integrated at the PBac{y+.attP-3B}VK0002-28E7 site. Cloning details are available upon request. Plasmid and BAC injection was performed by Bestgene (Chinmo).

The *Daam^GFP^* and *RhoGEF3^KO^* lines were generated using CRISPR-mediated HR. For each line, two gRNA oligonucleotides were cloned into pU6-BbsI-chiRNA (Addgene #45946) as described in addgene.org/crispr/OConnor-Giles/. Donor templates for HR were first produced by BAC recombineering in *E. coli* and then transferred into multicopy vectors as described in Venken et al. (2006). A BAC encoding *Daam* (CH321-32O15) was used to introduce sfGFP flanked by GVG linkers, at position 1087 of Daam-PA, i.e. 27 amino acids before the C-terminus. This position was chosen as a poorly conserved region between fly species. *Daam^GFP^* flies were viable with no phenotype. A partial deletion of the *RhoGEF3* gene was produced in the *RhoGEF3* BAC. The 3xP3-RFP selection marker (flanked by loxP sites) was produced by gene synthesis and inserted at the position of the deletion. Left and right homology arms flanking the target sites were 1.5 kb long. Proper HR was verified by genomic PCR.

Other stocks used in this study were: UAS-RhoGEF3dsRNA, par6-GFP, GFP-Baz, Numb-GFP (Couturier et al., 2013), Spider-GFP, neur-H2B-RFP, sqh-Cherry-MoeABD, sqh-MyoII-GFP, UAS-Cdc42 (BL-28873), UAS-Rac1 (BL-28874), UAS-Rho1 (BL-9390), GMR-Gal4, ap-Gal4 and tub-Gal80^ts^. Pictures of adult flies were taken on a AxioZoom V16 macroscope (Zeiss, Jena, Germany).

### Live imaging and image analysis

Live imaging of staged pupae and quantitative image analysis of Baz and Par6 asymmetry were performed as described earlier. Live imaging of mitotic SOPs was performed using a 63×/NA 1.4) objective (PL APO, DIC M27; Leica Microsystems, Wetzlar, Germany) on a Zeiss LSM780 microscope. SOPs were identified using a Histone2B-RFP expressed under the control of a *neur* cis-regulatory module (neur-H2B-RFP) or based on the polar distribution of Numb-GFP. For shape analysis (solidity), GFP-Baz movies were segmented as described in Besson et al. (2015). Cell contours were converted to Fiji polygon ROI and solidity values were calculated over a time interval preceding mitosis (∼14-15 h APF) using the corresponding Fiji plugin. Solidity values were represented as boxplots. For circularity at mitosis, segmented GFP-MoeABD movies were analyzed using the corresponding Fiji plugin. Cortical instabilities were scored blind in 4D (x,y,z,t) movies.

### Immunostainings

Staged larvae and pupae were dissected and stained following standard procedures. Primary antibodies were: rabbit and goat anti-GFP (1:1000; Abcam ab6673), mouse anti-Cut (1:500; clone 2B9, Developmental Studies Hybridoma Bank), guinea pig anti-Senseless (Nolo et al., 2000) (1:3000; a kind gift from H. Bellen, Baylor College of Medicine, Houston, USA), rabbit anti-aPKC (1:1000; sc216, Santa Cruz Biotechnology) and rabbit anti-Dia (Afshar et al., 2000) (1:1000; a kind gift from S. Wasserman, UCSD, San Diego, USA). F-actin was detected using atto-647 phalloidin (Sigma-Aldrich).

### Western blot

GFP-tagged RhoGEF3 was studied by western blot analysis using brain-disc complexes dissected from third instar larvae (five larvae per well). Protein extracts were prepared in 0.5% Triton buffer (50 mM Tris pH 7.4, 150 mM NaCl, 10% glycerol, 0.5% Triton X-100, 0.5 mM DTT, 1× protease inhibitor cocktail EDTA-free from Roche) and loaded on 4-20% precast Miniprotean TGX gels for SDS-PAGE. Proteins were transferred onto 0.2 µm Nitrocellulose membranes (BioRad). HRP-coupled anti-GFP antibodies (1:5000; Abcam, ab6663) were used to detect the different RhoGEF3-GFP isoforms with SuperSignal WestFemto (ThermoFisher Scientific).

### Protein production and *in vitro* GEF assay

The fragments encoding the C-terminal part of wild-type (EKN) and mutant (KAA) RhoGEF3-PA (513 amino-acids) were PCR-amplified from the *RhoGEF3* BAC (CH322-144N20) and cloned into the pGEX6P-2 plasmid (the EKN-to-KAA mutations were introduced in the BAC CH322-144N20 using BAC recombineering; cloning details available upon request). Following sequencing, plasmids were introduced in the BL-21 Rosetta strain for protein production. Recombinant proteins were purified on Glutathione sepharose beads (Amersham Bioscience). The RhoGEF3^EKN^ and RhoGEF3^KAA^ fragments were released from the beads by proteolytic cleavage using the prescission protease (GE Healthcare). Protein concentration was determined using the Pierce BCA Protein Assay Kit (ThermoFisher Scientific). The GEF activity of the purified RhoGEF3 fragments (0.8 µM) was tested using a fluorophore-based GEF assay that measures the uptake of the N-methylanthraniloyl-GTP (mant-GTP) by purified GTPases (2 µM) that were provided in the RhoGEF Exchange Assay Kit (Cytoskeleton Inc., Denver, USA) used for this assay (Leonard et al., 1994). We followed the manufacturer's protocol for 96-well plates. Fluorescence increase was measured on an Infinite M200 PRO spectrophotometer (Tecan, Mannedorf, Switzerland).

## Supplementary Material

Supplementary information
