# Supplementary material for: Regulation of cortical stability by RhoGEF3 in mitotic Sensory Organ Precursor cells in Drosophila
Source: Biol Open. 2017 Nov 3;6(12):1851–60. doi: 10.1242/bio.026641 (PMC5769646; doi:10.1242/bio.026641)
Supplement: Supplementary information [file biolopen-6-026641-s1.pdf]

Supplementary Figure

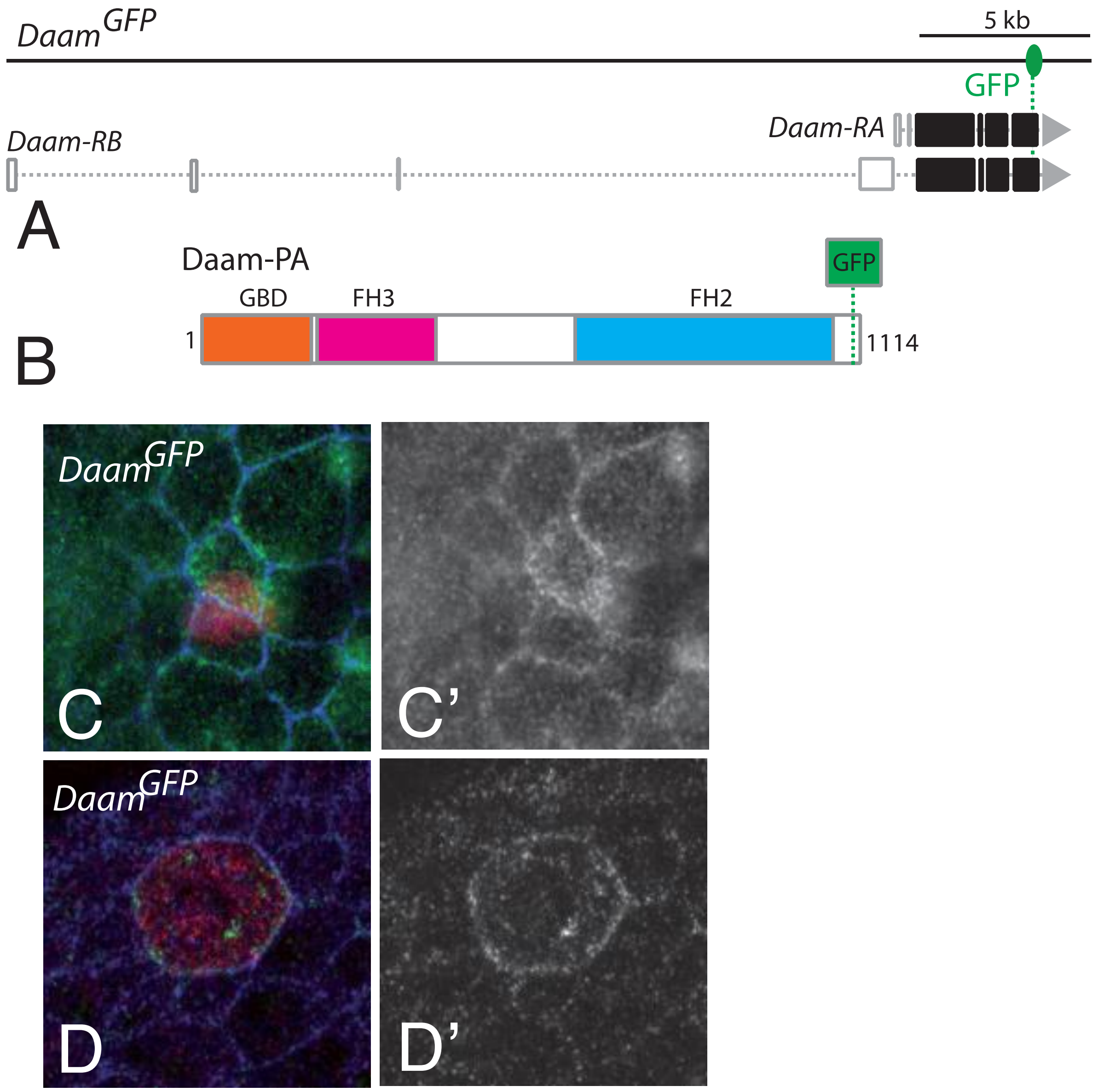

Figure S1: SOP-specific expression of a GFP-tagged Daam generated by CRISPR-HR

A) Genomic organization of the *Daam* locus showing only two transcript isoforms (RA and RB). Exons are shown as boxes (ORF in black). The point of insertion of the GFP (green) at the *Daam* locus is indicated. All isoforms are tagged. The position of the deletion in the *RhoGEF3*^{KO} flies produced by CRISPR-HR is indicated: most of the sequence encoding the RhoGEF3-PA was deleted. The 3xP3-RFP selection marker was inserted at the position of the deleted segment.

B) Domain structure of Daam-PA, encoded by the RA transcript isoform (A). The GTPase Binding Domain (GBD, orange) and the Formin Homology domains (FH2, magenta; FH3, blue) are shown. GFP (green) was fused at the C-terminus.

C-D') Daam-GFP (green) was detected at higher levels in SOPs (Senseless, red in C; Cut, red in D). Daam-GFP was detected at the apical cortex prior to division (C,C; aPKC, blue) and all around the cortex together with Dia (blue in D) at mitosis (D').

Supplementary Movies

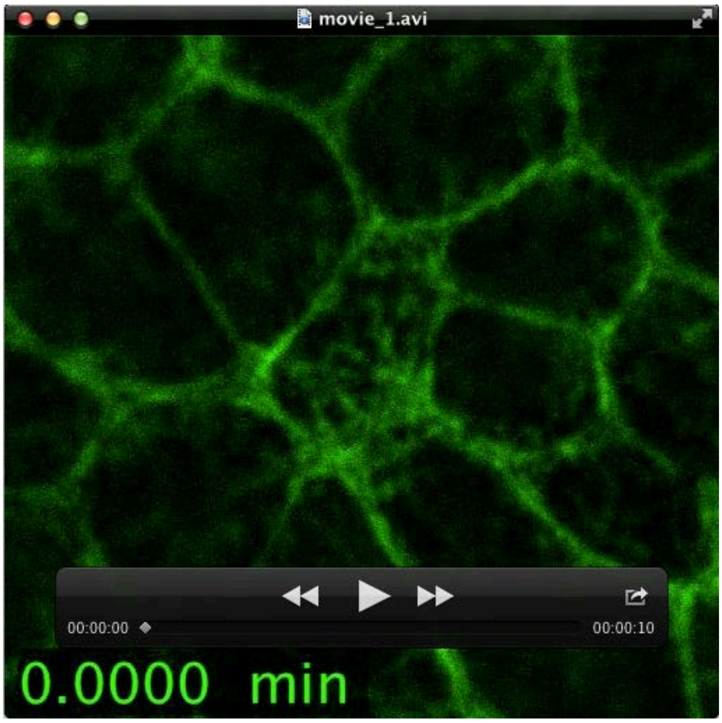

Movie 1: Live imaging of MyoII

Live imaging of GFP-tagged MyoII (green; time in min) in cells of the pupal notum. MyoII was mostly medial-apical in the SOP (at the center) and junctional in epidermal cells. Pulses and foci were observed in SOPs (note the cell shape deformation associated with the MyoII pulses).

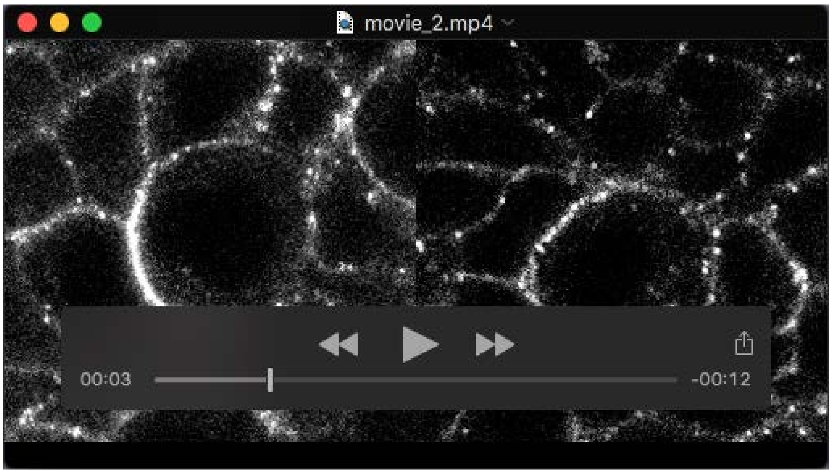

Movie 2: Live imaging of NumbGFP in wild-type and *RhoGEF3*^{RNAi} SOPs

Live imaging of GFP-tagged Numb in wild-type (left) and *RhoGEF3*^{RNAi} (right) SOPs. Cortical instabilities (arrow) were more frequently observed upon silencing of the *RhoGEF3* gene.

Biology Open • Supplementary information
